# Nationwide survey of treatment for pediatric patients with invasive fungal infections in Japan

**DOI:** 10.1007/s10156-013-0624-7

**Published:** 2013-06-04

**Authors:** Masaaki Mori

**Affiliations:** Department of Pediatrics, Yokohama City University Medical Center, 4-57 Urafune-cho, Minami-ku, Yokohama, 232-0034 Japan

**Keywords:** Nationwide, Survey, Questionnaire, Invasive fungal infections, Antifungal agents

## Abstract

In Japan, only a few antifungal agents have been approved for children, but in actual clinical practice, various antifungal agents used in adults are administered to pediatric patients with invasive fungal infections (IFIs). However, the pediatric dosages of some antifungal agents are not indicated in the package inserts or mentioned in the Japanese Mycology Study Group 2007 Guidelines for Management of Deep-seated Mycoses. We conducted a nationwide survey to determine how antifungal agents are being used to treat pediatric patients with IFIs in Japan. We sent a questionnaire to 792 medical centers that train pediatricians and received 250 (31.6 %) responses. In the past 5 years, 65 (26.0 %) of 250 facilities reported treating a total of 232 cases of IFIs. The characteristics of pediatric patients with IFIs were almost the same as adult patients except that immunological diseases and neonatal diseases are common as underlying diseases. Antifungal agents used in adults were all used in children. However, the dosages of some antifungal agents deviated from the package insert or guideline recommendations. As for the reasons for selecting a particular antifungal agent, strong antifungal activity (including potency, broad spectrum, and clinical efficacy) was favored over safety. These results can be used to revise guidelines for the management of children with IFIs.

## Introduction

Invasive fungal infections (IFIs) are a major cause of morbidity and mortality in immunocompromised patients [1–3]. IFIs often occur in children with various reasons for increased susceptibility to infections, including immature immune systems [4, 5]. Recently, the incidence of IFIs in children has increased with expanded pediatric use of therapies such as intensive chemotherapy, hematopoietic stem cell transplantation, or both for leukemia, and immunotherapy with steroids or immunosuppressants [6]. Early diagnosis of IFIs is challenging in children, not only because there are few characteristic symptoms in patients with severe underlying diseases, but also because of the invasive nature of diagnostic examinations for IFIs [7]. Although several new antifungal agents have been developed during the past decade, not all of these agents have been approved for children in Japan. Accordingly, the pediatric dosages of some antifungal agents are not indicated in their package inserts, and they are not mentioned in the Japanese Mycology Study Group 2007 [8] Guidelines for Management of Deep-seated Mycoses (Japanese guideline). We thought it was necessary to determine the actual status of antifungal agent usage in children.

The present survey was conducted to determine how antifungal agents are being used to treat pediatric patients with IFIs in Japan. The results can be used to revise guidelines for management of children with IFIs.

## Patients and methods

A questionnaire with the following three questions was sent to medical centers that train pediatricians in September 2009:

- (1-1) Have you treated pediatric patients with IFIs in the last 5 years?
- (1-2) If yes, which specific diseases?
- (2-1) Which antifungal agents do you use typically?
- (2-2) What is the usual dosage?
- (3) In general, what are the reasons for selecting a particular antifungal agent? Multiple answers were allowed from the following choices: (a) strong antifungal activity, (b) broad antifungal spectrum, (c) fungicidal activity, (d) no drug-resistant strains, (e) high clinical efficacy, (f) high clinical safety, (g) recommendation by a guideline, (h) abundant evidence, (j) indication in children, (k) considerable experience, (l) others.

The study protocol was approved by the Ethics Committee of Yokohama City University.

## Results

The questionnaire was sent to 792 medical centers that train pediatricians with 250 (31.6 %) replies. In the past 5 years, 65 of 250 (26.0 %) facilities reported treating a total of 232 IFI cases, whereas 185 facilities (74.0 %) reported no patients with IFIs. There was a variety of answers to the question about specific infections. They were classified into three categories, including underlying diseases, diagnoses, and causative organisms (Table 1). The underlying diseases for pediatric patients with IFIs included hematological diseases (25 patients), immunological diseases (9 patients), neonatal diseases (6 patients), and others (6 patients). Leukemia was the most common underlying disease, followed by congenital immunodeficiency diseases. Neonatal or premature status were also common. The major diagnosis of IFIs was sepsis (23 patients), followed by pneumonia (13 patients) and invasive pulmonary aspergillosis (10 patients). The main causative organisms were *Candida* spp. (25 patients) and *Aspergillus* spp. (25 patients). Nine (36.0 %) of 25 *Candida* spp. were further identified as *Candida parapsilosis* (3 cases), *Candida tropicalis* (3 cases), *Candida albicans* (2 cases), and *Candida glabrata* (1 case). None of the *Aspergillus* spp. were further identified.

**Table 1:** Characteristics of pediatric patients with IFIs

Underlying diseases	*N*	Diagnoses	*N*	Causative organisms	*N*
Hematological diseases	25	Sepsis	23	*Candida* spp.	25
Leukemia	13	Pneumonia	13	*Candida parapsilosis*	3
Malignant lymphoma	4	Invasive pulmonary aspergillosis	10	*Candida tropicalis*	3
Solid tumor	3	Brain abscess	4	*Candida albicans*	2
Febrile neutropenia	3	Meningitis	3	*Candida glabrata*	1
Hematopoietic stem cell transplantation	2	Disseminated candidiasis	3	Unidentified	16
Immunological diseases	9	Pulmonary mycosis	2	*Aspergillus* spp.	25
Congenital immunodeficiency disease	7	Spleen abscess	2	Unidentified	25
Hemophagocytic syndrome	1	Endocarditis	2	Zygomycetes	3
Systemic juvenile idiopathic arthritis	1	Pulmonary embolism	1	*Mucor* sp.	1
Neonatal diseases	6	Lung abscess	1	Unidentified	2
Extremely low birth weight infant	3	Intermuscular abscess in extremities	1	*Trichosporon beigelii*	1
Low birth weight infant	1	Arthritis	1	*Pneumocystis* sp.	1
Neonatal necrotizing enterocolitis	1			Unidentified	1
Perforation of the digestive tract	1				
Others	6				
Congenital nephrosis	1				
Peritoneal dialysis	1				
Peritonitis	1				
Acute encephalopathy	1				
Hypoxemia	1				
Near-drowning	1				

For each antifungal agent, questionnaire answers for the dose typically used for prophylaxis and treatment of IFIs in children were compared with recommendations for the diagnosis and treatment of pediatric patients with IFIs in the Japanese guideline [8] and the Infectious Diseases Society of America (IDSA) guidelines on aspergillosis (2008) [10] and candidiasis (2009) [11] (IDSA guidelines), as well as the dosages described in each package insert in Japan (Table 2).

**Table 2:** Usages and dosage of antifungal agents in pediatric patients

Antifungal agents	Usage	Facilities reporting use (I)	Dosage mean (range) (mg/kg)	Japanese guideline (mg/kg)	IDSA guidelines (mg/kg)	Dosage in Japanese package insert (mg/kg)
Micafungin	Prophylaxis	20	2.7 (1–4.5)			1
	Treatment	45	4.6 (1–7)	3–6	2–4	1–6
Fluconazole	Prophylaxis	40	6.4 (1.5–10)	3–6		12
	Treatment	42	8.9 (6–10)	10–12	6–12^a^	3–12
Fosfluconazole	Prophylaxis	40	6.7 (1.5–10)			
	Treatment	42	8 (4.5–10)			(FLCZ: 100–400 mg)^c^
Voriconazole	Prophylaxis	5	3.5 (3–6)			
	Treatment	39	7.6 (4–16)		7	(3–6)^c^
Itraconazole	Prophylaxis	22	4.2 (1–7.5)			(200–400 mg)^c^
	Treatment	14	6.2 (4–10)			(200–400 mg)^c^
Liposomal amphotericin B	Prophylaxis	2	2.3 (2–2.5)			
	Treatment	33	4.1 (1–5.5)		3–5^b^	(2.5–5)^c^
Amphotericin B deoxycholate	Prophylaxis	13	0.75 (0.75)			
	Treatment	10	0.8 (0.5–1)	0.25–1	0.5–1^a^	(0.25–1)^c^

^a^Recommended as primary therapy in neonatal candidiasis

^b^Recommended as alternative therapy in neonatal candidiasis

^c^The dosage for adults

### Micafungin

There were 20 facilities administering micafungin for prophylaxis, almost half of the 45 facilities administering it for treatment. The mean daily dose of micafungin used for prophylaxis was 2.7 mg/kg, higher than the package insert dosage. On the other hand, the mean daily dose of micafungin for treatment was 4.6 mg/kg, which was in accord with the Japanese guideline but was slightly higher than IDSA guidelines recommendations.

### Fluconazole

The number of facilities administering fluconazole for prophylaxis (40 facilities) and for treatment (42 facilities) was almost the same. The mean daily dose of fluconazole used for prophylaxis was 6.4 mg/kg, higher than the dosage recommended by the Japanese guideline. On the other hand, the mean daily dose of fluconazole used for treatment was 8.9 mg/kg, slightly lower than the Japanese guideline dose but in accordance with IDSA guidelines.

### Fosfluconazole

The number of facilities administering fosfluconazole for prophylaxis (40 facilities) and for treatment (42 facilities) was almost the same, as with fluconazole. The mean daily dose of fosfluconazole used for prophylaxis was 6.7 mg/kg, higher than the Japanese guideline recommendation. However, the mean daily dose of fosfluconazole used for treatment was 8.0 mg/kg, slightly lower than the Japanese guideline dose.

### Voriconazole

Voriconazole was most often administered for treatment (39 facilities), not for prophylaxis (5 facilities). The mean daily dose of voriconazole used for prophylaxis was 3.5 mg/kg. The mean daily dose of voriconazole used for treatment was 7.6 mg/kg, which was in accordance with IDSA guidelines.

### Itraconazole

The number of facilities administering itraconazole for prophylaxis (22 facilities) and for treatment (14 facilities) was almost the same, but not as high as fluconazole. The mean daily dose of itraconazole used for prophylaxis was 4.2 mg/kg. The mean daily dose of itraconazole used for treatment was 6.2 mg/kg.

### Liposomal amphotericin B

Liposomal amphotericin B was mostly used for treatment (33 facilities) rather than for prophylaxis (2 facilities). The mean daily dose of liposomal amphotericin B used for prophylaxis was 2.3 mg/kg. The mean daily dose of liposomal amphotericin B used for treatment was 4.1 mg/kg, which was in accordance with IDSA guidelines.

### Amphotericin B deoxycholate

The number of facilities administering amphotericin B deoxycholate for prophylaxis (13 facilities) and for treatment (10 facilities) was almost the same, but not as high as fluconazole and itraconazole. The mean daily dose of amphotericin B deoxycholate used for prophylaxis was 0.75 mg/kg. The mean daily dose of amphotericin B deoxycholate for treatment was 0.8 mg/kg, which was in accordance with both Japanese and IDSA guidelines.

The reasons for selecting a particular antifungal agent are shown in Table 3. The most frequent response was “strong antifungal activity,” followed by “broad antifungal spectrum” and “high clinical efficacy,” all of which were qualities related to antifungal activity. The next most frequent response was “high clinical safety,” which was followed by “considerable experience,” “recommendation by a guideline,” and “abundant evidence.” “Indication for children” was a very uncommon reason given.

**Table 3:** Reasons for selection of antifungal agents

Reasons	*N*
Strong activity	44
Broad spectrum	33
Fungicidal activity	11
No drug-resistant strains	4
High clinical efficacy	36
High clinical safety	30
Recommendation by a guideline	23
Abundant evidence	17
Indication for children	14
Considerable experience	26
Others	4
No response	8

## Discussion

We conducted a nationwide survey to identify how antifungal agents are being used to treat pediatric patients with IFIs in Japan. In our survey, 65 (31.6 %) of 250 facilities reported treating a total of 232 cases of pediatric IFIs in the past 5 years.

As in adults, hematological diseases were the predominant underlying condition in children, although immunodeficiency and neonatal or premature status were also common and unique to pediatric patients, compared to the data on visceral mycoses found at autopsy cases in Japan [11]. The major diagnosis of IFIs was sepsis, followed by pneumonia and invasive pulmonary aspergillosis. The main causative organisms were *Candida* spp. and *Aspergillus* spp., at approximately equal frequencies. The major diagnoses and causative organisms in children were also the same as for adults [11].

At the time of the survey (September 2009), only two antifungals, micafungin and liposomal amphotericin B, had been approved for pediatric use in Japan (fluconazole was approved in 2011). On the other hand, antifungal agents recommended by the Japanese guideline were fluconazole, itraconazole, amphotericin B deoxycholate, and micafungin [8], and the IDSA guidelines recommended fluconazole, voriconazole, amphotericin B deoxycholate, liposomal amphotericin B, and micafungin [9]. Our survey indicated that the antifungal agents not approved for pediatric use and not recommended by Japanese or IDSA guidelines were actually frequently used in clinical practice in Japan.

The antifungal agents most frequently used for prophylaxis were fluconazole and fosfluconazole, followed by itraconazole, micafungin, and amphotericin B deoxycholate. Dosages of fluconazole and fosfluconazole were slightly higher than those recommended by the Japanese guideline. This practice may result from the assumption that the causative organism could be a non-*albicans* *Candida* sp., which was substantiated by our observation that a non-*albicans* *Candida* sp. was more frequently identified than *Candida albicans* as the causative pathogen (Table 1). The dose of micafungin used for prophylaxis was higher than that recommended by the Japanese guideline, possibly from the assumption that *Aspergillus* spp. could be involved in IFIs.

The most frequently used antifungal agent for treatment was micafungin, followed by fuluconazole/fosfluconazole, voriconazole, and liposomal amphotericin B. Doses of micafungin and liposomal amphotericin B were in accordance with the Japanese and IDSA guidelines, respectively. The IDSA guidelines suggest 10–12 mg/kg micafungin for neonates [10]. In this survey, such a high dosage of micafungin was not reported. The doses of fluconazole (8.9 mg/kg) and fosfluconazole (8 mg/kg) were slightly lower than the 10–12 mg/kg recommended by the Japanese guideline for treatment, which is based on the assumption that 3–6 mg/kg fluconazole/fosfluconazole has been used for prophylaxis. Actually, if fulconazole or fosfluconazole is not effective, then it may be switched to other antifungal agents. The dose of voriconazole used by survey respondents was almost the same as that recommended by the IDSA guideline [10], where administration of 7 mg/kg voriconazole every 12 h is comparable to the adult dosage of 4 mg/kg every 12 h.

In this survey, the respondents seemed to consider antifungal activity more important than safety, probably because the conditions of pediatric patients being treated with antifungal agents were quite severe.

This survey suggests that the Japanese guideline needs to be revised. It also suggests that antifungal agents are commonly being used without approval for this age group. Both medical staff and pharmaceutical companies should make efforts to obtain the approval of all antifungal agents for pediatric use.
